# Incidence of pain after inguinal hernia repair in the elderly. A retrospective historical cohort evaluation of 18-years’ experience with a mesh & plug inguinal hernia repair method on about 3000 patients

**DOI:** 10.1186/1471-2482-13-S2-S19

**Published:** 2013-10-08

**Authors:** Marcello Donati, Giovanna Brancato, Angelita Giglio, Antonio Biondi, Francesco Basile, Angelo Donati

**Affiliations:** 1 Department of Surgical Sciences, Organ Transplants and New Technologies, General Surgery and Week Hospital Unit, University Hospital of Catania, Italy; 2 Department of Surgery, General and Oncologic Surgery Unit, Vittorio-Emanuele University Hospital of Catania, Italy

## Abstract

**Background:**

Chronic pain after prosthetic inguinal hernioplasty is one of the most important current issues in the current literature debate. Mechanisms related to pain development are only partially known. Influence of age as well as other factors is still unclear. The aim of this work was to evaluate whether development of chronic pain after open prosthetic plug and mesh inguinal hernioplasty is influenced by age.

**Methods:**

Analysis was retrospectively conducted, dividing our cohort of patients (2,902) who had undergone prosthetic open plug&mesh inguinal hernioplasty from Jannuary 1994 to May 2012, following only the age criterion (cut-off 65 yrs.), into two groups (Gr.A<65 yrs, Gr.B>65 yrs.). All patients were routinely submitted to a postoperative questionnaire. Complications such as analgesic assumption were registered in both groups. Pain intensity was classified following the Visual Analogic Scale (VAS). Incidence of chronic pain, discomfort, and numbness, was assessed in both groups. Statistical significance was assessed by X_2_-test.

**Results:**

Only 0.2% of patients suffered from a recurrence in our cohort. Postoperative chronic pain was observed in Gr. A in 0.12% of patients vs Gr.B 0.09% (*p*>0.05). Incidence of other postoperative symptoms such as discomfort or numbness were slightly prevalent on young patients (respectively *p *= 0.0286 and *p *= 0.01), while for hyperesthesia and sensation of foreign body no statistically significant difference of incidence between groups was observed.

**Conclusions:**

Real chronic pain after inguinal hernioplasty is a rare clinical entity. Other causes of chronic pain should be accurately researched and excluded. In young patients psychological factors seem to show a slight influence. There was no influence of age on chronic postoperative pain incidence after inguinal hernioplasty.

## Background

Chronic pain after prosthetic inguinal hernia repair is considered to be one of the unsolved problems after prosthetic inguinal hernioplsty [1,2]. Its incidence is referred to be from 0.03 to 31% in the scientific literature [3,4] (Table 1) many different mechanisms have been advocated to explain chronic pain after inguinal hernioplasty, and consequently different surgical solutions have also been proposed [5]. The aim of this work was to show the influence of age on incidence of postoperatively chronic pain after open prosthetic inguinal hernia repair in older patients (over 65 yrs.) retrospectively analyzing our single center 18 year experience with a published original mesh and plug technique [6,7] on 2902 consecutively operated patients.

**Table 1:** Incidence of groin pain after inguinal hernioplasty reported in the literature

Author	Year	Journal	N° cases	Followed up	Chronic Pain (%)
Amid PK	1996	Eur J Surg	4000	3480	0.03 %
Negro P	2000	Chir Ital	839	764	0.9 %
Courtney CA	2002	Br J Surg	5506	4062	3 %
Heikkinen T	2004	Surg Endosc	61	59	7%
Benedetti M	2005	Hernia	685	?	11.9%
Haapaniemi S	2002	Eur J Surg	272	223	15 %
Leardi S	2003	Chir Ital	100	60	25%
Callesen T	1999	Br J Surg	466	419	25 %
Kumar S	2002	Br J Surg	560	454	30 %
Koninger J	2004	Langenbecks Arch Surg	93	76	31%
Inaba T	2012	Surg Today	219	191	14.7%
Birk D	2013	Hernia	220	220	1.2%
Bittner R	2011	World J Surg	300	300	3%
Bright E	2010	World J Surg	9607	?	4.9%

## Materials and methods

From January 1994 to May 2012, in our unit, 2902 patients were submitted to an open prosthetic inguinal hernioplasty for primary inguinal hernia following our previously described plug and mesh procedure [6,7]. Most of them received the surgical treatment in a day surgery setting [8,9] as for other kind of hernias in our center [10,11]. They were 2752 males (94.5%) and 150 females (5.5%).

All patients were preoperatively informed about the procedure, anesthesia, intraoperative feelings or sensation during surgical manipulation and postoperative care as previously described [8,9]. We divided the above mentioned collective into 2 Groups based on age: group A (<65 yrs: 1795 patients) and group B(>65 yrs:1107 patients). Group A showed a comorbidities in only about 44.8% (805/1795) of patients. While in group B 938 patients showed comorbidities (84.7%), 478 (50.9%) of them showing were relevant (ASA III-IV) (Table 2).

**Table 2:** Patients comorbidities in both groups

	**Group A <65 yrs**. (1795 pts.)	**Group B >65 yrs**. (1107 pts.)
Cardiovascular	437	449
Respiratory	122	131
Metabolic	105	102
Hematologic	41	8
Neurologic	38	7
Others	62	241
Total	805 (44.8% of cohort)	938 (84.7% of cohort)

Comorbidities of both groups are summarized in table 2. All patients underwent the above mentioned surgical procedure, most of them under local anesthesia (as showed in Table 3), following renowned and well described methods used by our group for many years also for other kinds of hernias [12,13].

**Table 3:** Type of anesthesia for herniorrhaphy and day surgery distribution in both groups

	**Group A <65 yrs**. (1795 pts.)	**Group B >65 yrs**. (1107 pts.)
Local Anesthesia	1775 (98.8%)	1081 (97.5%)
Spinal Anesthesia	0	11 (1%)
General Anesthesia	20 (1.2%)	16 (~1.5%)
Day Surgery	1730 (96.4%)	889 (80.3%)

Groups were evaluated for incidence of postoperative (defined as up to 12 postoperative day) and delayed (chronic) pain after inguinal hernioplasty as well as other sensations/complains such as referred numbness, discomfort, hyperestesia, sensation of foreign body.

The operation was conducted under local, spinal and general anesthesia in Group A respectively in 98.8%,0 and 1.2% of patients, while in Group B in 97.5%,1% and 1.5%. No statistically significant differences regarding distribution of patients in local general and spinal anesthesia were found between the 2 groups (Table 3).

Patients were asked about pain intensity on postoperative recovery and during follow-up checks by using a Visual Analogic Scale (VAS). Then we followed-up the patients for the about 2 weeks after the operation, by collecting data about daily activities, referred pain during the daily activities, type of pain, duration of pain, assumption of painkillers. Pain intensity when present was classified following the Visual Analogic Scale (VAS) of Glasgow ranging from 0-10, as previously used in other reports [10,14]. All patients were clinically evaluated together with an ultrasound follow-up after 3 months and after 1 year [15], in order to establish presence of recurrence, ask residual pain, other minor symptoms (like numbness, hyperestesia etc..etc..) and eventually chronic pain (we defined chronic pain as the presence of pain for more than 6 months after the operation). All differences were statistically evaluated through the X_2_-test for significance (*p < 0.05*).

## Results

We had a total of 6 recurrences (0.2%) in our cohort: 4 really inguinal hernia recurrences and 2 pseudo-recurrences as 2 becoming symptomatic after hernioplasty (misdiagnosed hernias).

In group A 57.2% of patients used analgesia during recovery and 36.9 at home while in group B respectively 48.2 and 34.3%.

After one year In group A no more pts referred light pain in both groups, rarely they referred mild pain (VAS 4-7) without differences between young and old patients, so like for strong chronic pain (VAS 8-10). Minor symptoms like discomfort and numbness showed a slight prevalence on Gr.A in front of Gr.B after one year (*p *= 0.0286 and 0.01). Table 4.

**Table 4:** Distribution of symptoms in both groups after 1 year

	**Group A <65 yrs**. (1795 pts.)	**Group B >65 yrs**. (1107 pts.)	*p*
Discomfort	308 (17.1%)	156 (14.1%)	***0.0286***
Foreign Body Sensation	215 (11.9%)	132 (12%)	0.965
Numbness	157 (8.7%)	68 (6.1%)	***0.01***
Hyperesthesia	130 (7.2%)	70 (6.3%)	0.34
Burns	0	0	-
Light pain*	0	0	-
Mild Pain**	5 (0.28%)	2 (0.18%)	0.6
Strong Pain***	2(0.12%)	1(0.09%)	0.8

*VAS=Visual Analogic Scale. Pain is here intended to be continous*.

* 0-3 in VAS

** 4-7 in VAS

***8-10 in VAS

During recovery in group A early postoperative pain till 2 weeks, was more often observed than in group B: 17.1% vs 14.1% *p *= 0.009. While delayed postoperative pain and chronic pain showed no significance on incidence in both groups: *p *= 0.64 and *p *= 0.836 (Table 5). Also for intermittent and rare pain (often due to changes in humidity) no differences weres found *p *= 0.136. A total of 7 patients in the collective of patients complained middle pain whil only 3 strong pain. In this 3 of a vertebral compression or coxo-femoral arthrosis was identified as a secondary cause of inguinal pain by MRI, and they were consequently treated. The other 7 patients reporting middle pain (VAS 4-7) referred a progressive relief/reduction of symptoms not hesitating to begin with legal proceedings, were treated conservatively without any exploration of the groin but with local infiltration of local anesthetic drugs.

**Table 5:** Temporally-related distribution of pain in both groups

	**Group A <65 yrs**. (1795 pts.)	**Group B >65 yrs**. (1107 pts.)	*p*
Early postoperative pain* (VAS>4)	484 (26.9%)	251 (22.6%)	***0.009***
Postoperative pain (after 2 weeks until 30 days) (VAS>4)	287 (16%)	149 (13.4%)	0.064
Rare pain (not continous) (VAS<4) (after 6 months)	628 (34.9%)	418 (37.7%)	0.136
Chronic pain (continous) (VAS>7) (after 6 months)	2 pts. (0.12%)	1 pts. (0.09%)	0.837

* any pain despite analgesics assumption.

## Discussion

Chronic pain after inguinal hernioplasty can be defined as a persistent pain in operated groin region, lasting from 6 months after reoperation until at least one year, following EHS guidelines definition [1,16]. Of course for patients post-hernioplasty pain begins after the operation and they have fear to have a chronicization of pain, when its duration is more then one week. However, previous studies have shown the influence of many factors.

The incidence in world literature is estimated between 1-31% (Table 1). This large range maybe reflects heterogeneity of surgical approaches and techniques, duration of follow-up and interpretation of data such as referred symptoms from each Author.

Factors related to surgical techniques, psychological factors [17], patients’ fear, and open or laparoscopic approach were previously analyzed to assess the influence on postoperative chronic pain[1]. Mechanisms (nerve entrapment, nerve trauma, amputation neurinoma, cicatrization damage) were advocated responsible for chronic pain after inguinal hernioplasty and were previously investigated in other papers [2]. The real incidence of chronic pain is lower than usually reported, discrepancies in published data are mainly due to the definition of "pain". Most patients’ complaints are really just discomfort and also in our experience, as elsewhere reported, really severe chronic pain is extremely rare [18]. In previous publications other authors reported the influence of age as a risk factor for developing chronic inguinal pain after hernioplasty [1,19,20]. Even if to date, to our knowledge, no specific studies have been published assessing the influence of age alone on post-herniorrhaphy chronic pain, it is commonly accepted that young age is a risk factor for chronic pain [16]. In our retrospective analysis we could not confirm such previous findings; on the contrary we found there was no statistical significance in the difference between our two groups (younger and older patients) except for early postoperative pain. In young patients a little prevalence of minor symptoms like numbness or discomfort was also found, this could maybe related to a more active life and also to psycological/emotional reasons. We interpreted these findings with the idea that maybe other factors, rather than age, have a greater influence on triggering chronic pain. As we stated in other papers, our surgical method presupposes identification and preservation of all three nerves of the inguinal region (Ileo-inguinal, ileo-ipogastric and genito-femoral). As reported in a previous study the preventive identification of all three nerves is a factor reducing the risk of post-operative chronic pain [21,22]. Another study reported controversial data about this issue, while Amid confirmed that the identification of structures is the first preventive factor to avoid post-operative complications [23]. Even other technical details such as mesh fixation showed an influence on pain. The TIMELI trial has shown a reduced risk of post chronic pain by using fibrin sealant as a fixation method of mesh instead of common stitches [24], this was also confirmed in a recent metanalysis [25]. Even the approach (open or laparoscopic) has been advocated as a risk factor for chronic pain [26,27], however, there are same data in contrast with this assumption [28] especially in laparoscopic techniques, mesh fixation methods seem to be of capital importance [29,30]. Our technique does not fix the mesh to the abdominal wall as it is self-adhesive, this could explain the low incidence of post-operative chronic pain [31].

Mesh weight was also introduced as a risk factor for development of chronic pain, thus light-weight meshes were introduced to reduce it [32]. However, further studies have shown that even if early pain is reduced by light-weight meshes, in long term follow-ups this difference is reduced [33]. A recent study has shown no influence of mesh weight in the incidence of chronic pain when the operating surgeon and the surgical technique are the same [34]. Preventive resection of all three nerves was also proposed [35,36] even if its effectiveness in combination with open mesh procedures remains questionable [37].

Over time (more than 10 years) patients tend to refer pain tolerance until disappearance or relief of symptoms [38]. Thus in cases of light-mild pain a surgical exploration could be avoided. Minimal contact between mesh and nerves, use of self-adhesive meshes, routine identification of nerves, a correct pre-operative flow-chart excluding other causes of inguinodynia, psychological pre-operative care of patients with detailed information about the operation could explain the low chronic pain rates in our series.

Treatment methods in literature range from local drug infiltration using desametasone or steroid-like drugs to anesthetics such as bupivacain [39]. Radiofrequency ablation of nerves was also described and tested [40]. Laser therapy such as TENS was also tested as well as physical therapy. Surgical exploration of the groin with excision of all three nerves (neurectomy) [41] is intended to be the „*ultima ratio*" of treatment and has been effective in many series [42-44], even if in long-term follow-up a relapse of symptoms is possible in a not insignificant percentage of patients [45-48]. In our series it was not necessary to surgically manage chronic pain. All three patients complaining strong pain obtained relief of symptoms after undergoing a vertebro-lumbar operation for discal hernia or orthopedic operation for coxo-femoral arthrosis diagnosed after inguinal hernia repair by an additional diagnostic flow-chart. The other group experiencing middle pain, knowed a progressive reduction of symptoms over time.

## Conclusions

In conclusion our data show that age is not a risk factor to developing chronic inguinal pain after hernioplasty. Real chronic pain is to be considered a rare entity. Unfortunately a heterogeneous group of other sensations more frequently affects patients after prosthetic inguinal hernioplasty, not really affecting daily activities and quality of life. Other causes of chronic pain should be carefully excluded before defining symptoms as chronic pain after hernioplasty.

Even if pain in younger patients has the same incidence as in older patients, in younger patients it is less well tolerated and they are prone to a greater analgesic assumption, additionally early postoperative pain in younger patient is more frequent. In the elderly all other sensations are better tolerated. A precise surgical technique, with systematic identification and preservation of inguinal region nerves, should be considered an important factor to prevent chronic pain. A conservative treatment, in many cases, is possible, surgical re-exploration of the groin with total neurectomy of all three nerves should be carefully considered as „*extrema ratio*" of treatment.

## List of abbreviations used

VAS (Visual Analogic Scale); MRI (Magnetic Resonance Imaging); EHS (European Hernia Society); TENS (Transcutaneous Electrical Nerve Stimulation).

## Competing interests

The Authors have no conflict of interests. The Authors received no financial support for the present study.

## Authors' contributions

MD:designed the study, co-collected data, wrote the paper, made literature research, gave final approval of the version to be published. GB: co-wrote the paper and co-made literature research, gave final approval of the version to be published. AG: collected and interpreted data, gave final approval of the version to be published. AB: co-collected data and draft tables, gave final approval of the version to be published. FB: made finale revisions, gave final style to the paper, gave final approval of the version to be published. AD: conception and co-designed of the study, made revisions, gave final style to the paper, gave final approval of the version to be published.

## Authors information

MD: Assistant Professor of Surgery at University of Catania. GB: Associate Professor of Surgery at University of Catania. AG: Resident in General Surgery at University of Catania. AB: Associate Professor of Surgery at University of Catania. FB: Full Professor of Surgery at University of Catania. AD: Full Professor of Surgery at University of Catania.

## Declarations

Funding of this article came from University funds.

This article has been published as part of *BMC Surgery *Volume 13 Supplement 2, 2013: Proceedings from the 26th National Congress of the Italian Society of Geriatric Surgery. The full contents of the supplement are available online at http://www.biomedcentral.com/bmcsurg/supplements/13/S2
